# Attitudes of Healthcare Workers toward Influenza Vaccination in the COVID-19 Era

**DOI:** 10.3390/vaccines10060883

**Published:** 2022-05-31

**Authors:** Tommaso Sani, Ilaria Morelli, Donatella Sarti, Giovanni Tassinari, Maria Capalbo, Emma Espinosa, Beatrice Gasperini, Emilia Prospero

**Affiliations:** 1 School of Nursing, Università Politecnica delle Marche, Via Lombroso, 61122 Pesaro, Italy; to.sani@outlook.it (T.S.); illy.morelli@gmail.com (I.M.); 2 Department of Biomedical Sciences and Public Health, Section of Hygiene, Preventive Medicine and Public Health, Polytechnic University of Marche, 60126 Ancona, Italy; sarti.donatella05@gmail.com (D.S.); e.prospero@univpm.it (E.P.); 3 General Direction, Azienda Ospedaliera Ospedali Riuniti Marche Nord, 61122 Pesaro, Italy; giovanni.tassinari@ospedalimarchenord.it (G.T.); maria.capalbo@ospedalimarchenord.it (M.C.); 4 Geriatric Unit, Azienda Ospedaliera Ospedali Riuniti Marche Nord, 61032 Fano, Italy; e.espinosa@univpm.it

**Keywords:** influenza, vaccination, HCWs, COVID-19

## Abstract

Healthcare workers (HCWs) are reluctant to participate in the influenza vaccination program, despite their high risk to contract and diffuse influenza due to professional exposure. The onset of the COVID-19 pandemic could raise HCW flu vaccination adherence. The aim of this study was to assess HCW attitudes toward influenza vaccination in the COVID-19 era. A multicenter observational study was carried out in three Italian hospitals (two in Pesaro and one in Fano, Marche region, Italy). Data about HCW influenza vaccination between 2013 and 2021 were extracted from the vaccination registers. An online questionnaire was sent to HCWs from July to October 2020 to assess their opinion about influenza vaccination in terms of knowledge, attitude, and practice during the COVID-19 pandemic. The number of flu-vaccinated HCWs increased from 3.7% in the 2013–2014 flu season to 53.6% in the 2020–2021 flu season (*p* < 0.001). About 15% (*n* = 324) of HCWs responded to the online questionnaire, and 30.5% of them declared that they had changed their minds on flu vaccination after the COVID-19 pandemic, deciding to get vaccinated. The COVID-19 pandemic significantly increased HCWs’ attitudes toward flu vaccination. However, flu vaccination adherence remained low and should be improved.

## 1. Introduction

Vaccinations are one of the most effective public health interventions to prevent the spread of infectious diseases. Influenza is a global health issue, and it causes significant mortality, in particular among elderly and high-risk groups [1,2]. Influenza transmission among healthcare workers (HCWs) is widely known. Indeed, HCWs are at risk of occupational exposure due to contact with patients who may carry the virus and to be a vector of transmission to vulnerable patients [3,4,5]. HCWs are a priority group for seasonal influenza vaccination to protect themselves and improve patient safety. Nevertheless, flu vaccination rates among HCWs are low in most of western countries, ranging from 5% to 53% [6].

Data on HCWs’ flu vaccination coverage are not available in Italy for the whole country; however, single local studies report coverage rates of 5–34% in different regions and hospitals [7,8,9,10,11,12]. Several factors affect flu vaccine uptake in Italy: social determinants, gaps in knowledge on infection risk and vaccine safety and efficacy, and inadequate communication from the relevant public health institutions during vaccination campaigns [13,14,15,16,17]. Some studies show that the main reasons for non-vaccination among HCWs are concerns about possible side effects, such as allergic reactions or infection, doubts of vaccine efficacy, beliefs of not being at risk of infection, or considering influenza a mild disease [15,16,17].

Promotion campaigns are very important to improve influenza vaccination uptake among HCWs. They can include multiple approaches, such as onsite educational visits (OEV), forum theater, communication tools using posters in frequently visited areas, information factsheets (printed and online), and banners on the hospital’s intranet [18,19,20]. A recent review of these strategies shows that they can achieve a substantial increase in vaccination coverage, even if it does not reach satisfactory levels (<40%) [21].

During the 2020–2021 flu vaccination campaign, there was a greater awareness of flu vaccination importance among HCWs because of possible co-circulation of the influenza and SARS-CoV2 viruses and because they are the most exposed to contract COVID-19 infection [22,23]. A retrospective study showed that influenza vaccination attitudes have changed during the COVID-19 pandemic among Italian HCWs in cancer centers (Emilia Romagna region), increasing their adherence to flu vaccination [24].

The aim of this study was to assess changes in the attitudes of healthcare workers toward flu vaccination from 2013 to 2021 in the COVID-19 era in three Italian hospitals in the Marche region.

## 2. Materials and Methods

This was a retrospective observational study carried out on HCWs’ flu vaccination attitudes and coverage in three Italian hospitals: San Salvatore Hospital (Pesaro, PU), Muraglia Hospital (Pesaro, PU), and Santa Croce Hospital (Fano, PU). All hospitals were located in the province of Pesaro Urbino in the Marche region, Italy. Each hospital has about 100 beds and belongs to the same hospital company (Azienda Ospedaliera Ospedali Riuniti Marche Nord, Pesaro, Italy), which was created with regional law n. 21/2009 in the Marche region (Italy) A vaccination register has been filled each year since 2013 in order to trace the number of vaccinated HCWs in all three hospitals. The director of the hospital company approved the study. However, due to the retrospective analysis and the anonymity of the questionnaire and data protection strategies, a formal approval by the ethical committee of the Marche region was waived.

### 2.1. Seasonal Vaccination Program

The vaccination program was performed from November to January, according to the World Health Organization (WHO) recommendations [25]. The flu vaccination for HCWs was freely available upon request.

A promotion campaign was conducted to inform all HCWs about flu vaccination before the start of vaccine administration in November. Leaflets were exposed in each ward, posters were distributed in the most frequented hospital areas, and the head nurse contacted each HCW to propose the vaccination. This strategy has been the same since 2013, when the vaccination registry was started.

However, the promotional campaign was intensified in 2019–2020. That campaign was performed from February to May 2020 due to the low rate of adherence to the vaccination program. Promotion interventions included lectures, intranet presentations, and posters on flu vaccination. Lectures were conducted during company training hours describing the advantages of flu vaccination and discussing the answers to the most frequent doubts (such as type and incidence of side effects) and the main reasons why it is important to get vaccinated. Usually, the vaccination promotion campaign started in September; however, the hospital director decided to start the vaccination campaign earlier in 2020 due to the COVID-19 pandemic. This decision was forced by several reasons: (1) to avoid a possible co-infection with COVID-19 and influenza; (2) to reduce as much as possible the HCWs’ absence from work due to illness; (3) to facilitate the differential diagnosis between COVID-19 and the flu because they have similar clinical manifestations; and (4) to reduce hospital pressure and the overcrowding due to admissions for influenza.

The promotion of the flu campaign was strongly encouraged by the Italian Ministry of Health and the Hospital director followed this suggestion.

### 2.2. The Questionnaire

An online electronic survey was sent to all HCWs of the three hospitals in study from July to October 2020. The informed consent was included in the electronic questionnaire. The questionnaire aimed to understand HCWs’ opinions about flu vaccination and how it was influenced by the SARS-CoV2 outbreak. HCWs were asked to score, on a Likert scale from 4 (very in agreement) to 1 (do not agree), a series of statements assessing knowledge and opinion about vaccination, which are reported in Table 1. The questionnaire also investigated the reasons for vaccination refusal. In particular, HCWs were asked to select among a list of possible concerns, which are reported in Table 2. Concerning the COVID-19 pandemic’s influence on HCWs’ opinions about flu vaccination, they were asked to answer yes/no to a set of questions, which are reported in Table 3. The targeted HCW categories were: physicians, nurses, obstetricians, radiologist technicians, socio-sanitary care assistants, other healthcare providers, and non-healthcare providers (office-based personnel).

### 2.3. Statistical Analysis

Frequencies and percentages of influenza vaccination were calculated for each year from 2013 to 2021. The data were analyzed, and graphs and tables were produced to obtain a complete view of influenza vaccination adherence from 2013–2014 to the 2020–2021 flu season. A descriptive analysis was carried out to describe the sample, and results are presented as frequencies and percentages. The chi-square was used to compare the frequencies. A univariate analysis and a logistic regression were performed to assess a possible association between HCWs characteristics (age, gender, years of employment, hospital role, and setting of employment) and the adherence to the flu vaccination in 2020. Statistical significance was set at *p* < 0.05. The statistical analysis was conducted using SPSS version 25 (Chicago, IL, USA).

## 3. Results

During 2020, there were 2021 HCWs employed in the hospital company. Physicians, nurses, and the other healthcare providers have hospital e-mail addresses. The questionnaire was sent to the hospital e-mail addresses, which are usually used for the hospital company communications. Among 2021 HCWs of the three hospitals in study, 324 accepted to fill the questionnaire (response rate of 15.3%). They included 258 (79.6%) females and 65 (20.1%) males, and their median age was 48.1 years (30–68 range). Concerning the HCW categories, 133 (41.333%) were nurses, 99 (30.6%) were physicians, 23 (7.1%) were healthcare professionals, 20 (6.2%) were nurse coordinators, 22 (6.8%) were technicians, and the remaining 25 (7.8%) included physiotherapists, administrative personnel, obstetricians, psychologists, biologists, dieticians, social assistants, and chemists. The number of years of employment and the setting where they were working (medical wards, surgical wards, ambulatory care, and no contact with patients) are also described and presented in Table S1.

**Figure 1:**
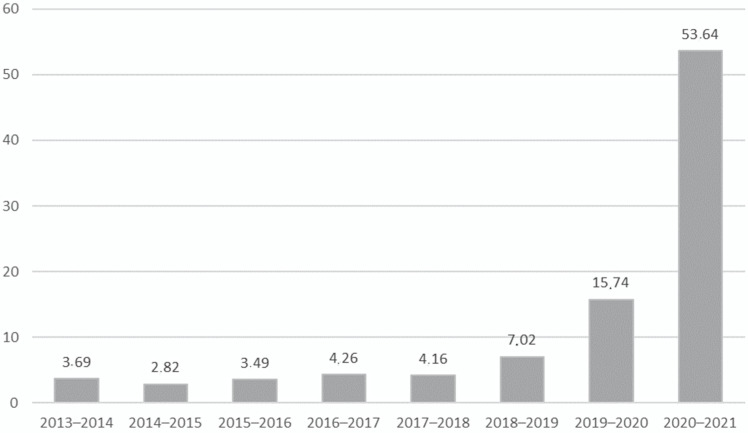
Number of vaccinated HCWs from 2013–2014 influenza season to 2020–2021.

Overall, HCW adherence (total HCWs of the three hospitals) to flu vaccination rose from 3.7 in the 2013–2014 season to 53.6% in 2020–2021 (*p* < 0.001). The median percentage of vaccinated HCWs was 3.7% (2.8–4.3% range) from 2013/2014 to the 2017/2018 flu season. The rate of vaccinated HCWs increased in the following flu season from 4.2% in 2017/2018 to 7% in 2018/2019 and 15.7% in the 2019–2020 flu season (*p* = 0.001). The main increase in the vaccinated HCW rate was observed after the COVID-19 pandemic. In particular, 53.6% of HCWs were vaccinated against influenza in the 2020–2021 flu season (*p* < 0.001) (Figure 1).

The responses of HCWs about the knowledge and opinion on vaccinations are presented in Table 1.

Most HCWs (94.1%) agreed that: vaccination was important to reduce/eliminate even serious diseases; serious adverse reactions from the vaccine were very rare (72%); stopping the vaccination would result in the return of rare infection (92.5%), and HCWs were available to face doubts or perplexities about vaccinations (72.6%). However, only 58.9% of HCWs claimed to be prepared and updated on vaccinations.

Some HCWs declared that serious side effects from the vaccine were hidden (18.9%), and that the disease was less dangerous than the vaccine itself (16.6%). One in five thought that those who do not vaccinate/get vaccinated were blamed by National Health System operators (22.8%).

HCWs were aware that influenza was one of the diseases most easily transmitted by health professionals (94.1%) and that influenza vaccination was recommended for HCWs by the Ministry of Health in the 2019–2020 flu season (99.4%). However, only 69.5% knew that the circular of the Ministry of Health, *Prevention and control of influenza: recommendations for the 2020–2021 season*, expanded the range of the population for which vaccination was recommended and free.

Concerning the motivations to refuse vaccination, answers are reported in Table 2. The most reported reason for vaccination refusal was concern about possible adverse events (16.8%). A small percentage of HCWs believed that the vaccine was not effective (14.6%) or was more dangerous than the disease itself (14.6%).

Other motivations were: a lack of knowledge of vaccination points (13.5%), a belief that vaccines were an economic business of pharmaceutical companies (11.2%), a belief of having low chances of contracting the disease (10.1%), or having no worries about the consequences of the infection (10.1%).

Predictors of vaccine refusal in 2020 did not emerge from the HCWs’ characteristics available in our dataset. However, HCWs working in medical wards or in ambulatory care and physicians seemed to be more prone to be vaccinated than other HCW categories (Table S2).

After the COVID-19 pandemic, 30.5% of HCWs declared they have changed their opinion on flu vaccination, and 33.9% decided to get vaccinated. A low percentage of HCWs (1.6%) did not want to get vaccinated (Table 3).

Concerning the information on COVID-19 pandemic, HCWs knew the health information from the Ministry of Health (94.7%) and consulted the ECDC and/or WHO web pages (41.4%) in the 2019–2020 flu season.

The majority of HCWs (69.5%) were aware of the publication of the Circular of the Ministry of Health, *Prevention and control of influenza: recommendations for the 2020–2021 season*, which expanded the range of the population for which vaccination was recommended and free. Among HCWs, 29.9% indicated the influenza vaccination for the 2020–2021 season would have been useful to reduce the number of influenza cases among HCWs and to facilitate the diagnosis and management of suspected cases because of the similar symptoms between COVID-19 and the flu. They believed that the effect of the pandemic on vaccinations would result in a vaccination increase (30.5%).

## 4. Discussion

Influenza has a high impact on the population in terms of morbidity and mortality, and notwithstanding the current International and European guidelines, which strongly recommend seasonal flu vaccination for healthcare workers, their vaccination coverage rates are still very low in Italy [11,12].

Many studies report that the main reason for HWCs’ flu vaccination refusal is correlated to socio-demographic and professional factors, such as age, gender, and professional role [15,16,17]. However, an overall higher confidence and awareness among younger HCW employees, such as medical residents/students and trainees was observed in the last decades, resulting in higher vaccination coverage rates [14]. A significant increase in HCWs’ vaccination rate was observed after the spread of COVID-19. However, the adherence to the flu vaccination campaign remained low.

Previous Italian data reported an overall HCW flu vaccination rate of 20.8% [26,27]. Different factors determine a low compliance to flu vaccination that is often associated with a lack of promotion campaigns. Vaccination is the only reliable protection against nosocomial infection transmission. Vaccination promotion campaigns are needed to improve coverage rates [2,18,19,20,21,28,29]. The knowledge on influenza is very important, and HCWs have a key role in vaccination promotion [26]. Flu vaccine uptake among the population, moreover, seems to be influenced by regional vaccination strategies [14]. Flu vaccination multimedia promotion campaigns (letters in pay slip and posters) are effective in increasing the number of vaccinated HCWs (7.6% vs. 5.6%, *p* < 0.005) and their intention to get vaccinated in the future (13.1% vs. 36.6% *p* < 0.005) [30]. The use of different strategies, including lectures, intranet, and posters may be useful to improve the adherence of HCWs to flu vaccination. In our study, only 58.9% of HCWs claimed to be prepared and updated on vaccinations [18,19,20,21].

The influenza vaccination is not mandatory in Italy. However, during the past years, the vaccination campaign has been strengthened throughout the national area [18,19,20,21]. In our hospitals, a campaign for the promotion of the flu vaccination has been strongly reinforced since 2019. The number of vaccinated HCWs increased (3.7% vs. 15.7%, *p* < 0.001) from the 2018–2019 season to the 2019–2020 flu season, indicating an improvement, although not sufficient, in the awareness of health workers.

A significant increase in the adherence to the flu vaccination was also observed in 2020–2021 flu season (53.4%, *p* < 0.001), suggesting that the COVID-19 pandemic had a decisive influence on flu vaccination adherence. Indeed, after the COVID-19 pandemic outbreak, 30.5% of employees declared that they had changed their minds about flu vaccination. Of note, during our study, several HCWs were directly involved in the care of COVID-19 patients because Pesaro Province had the highest number of COVID-19 cases in Marche at the beginning of the pandemic.

The increase in the HCW vaccination rate may depend on several factors. First, the vaccination campaign was improved and strengthened during 2020. Second, the hospital direction decided to improve vaccine accessibility, delivering vaccine doses in each hospital department. This strategy posed some organizational issues, and an important effort was made to maintain a strict control over the number of doses and the number of HCWs who got vaccinated. However, it ameliorated the possibility to accept the flu vaccination. Third, the public opinion and the health campaign promoted by the Italian Ministry of Health contributed to increase the awareness of the importance of flu vaccination during the COVID-19 pandemic.

The increase in HCWs flu vaccination coverage rate seems to be consistent with data reported in different countries for other risk groups. During the 2020/21 flu season, the elderly population’s influenza vaccination rates increased in England (+8.5%), France (+7.9%), Israel (+8.4%), Italy (+10.7%), the Netherlands, (+6.6%), the Philippines (+3.0%), Poland (+3.3%), Spain (+13.0%), and the United States (USA) (+5.4%) in comparison to the previous season [31].

The increase in willingness to receive the flu vaccination during the COVID-19 pandemic was also reported in another Italian study, with 68% of respondents reporting a willingness to receive a flu vaccination in the 2020/21 season, with 95% of those ever vaccinated and 45.8% of those never vaccinated in the previous six flu seasons [14].

A small percentage of the HCWs in the 2019–2020 flu season had doubts about the honesty of vaccine manufacturers and believed that the vaccine was unsafe and was more dangerous than the disease, denied the possibility of the return of rare diseases kept under control by vaccination, and believed that vaccination can be substituted by the mere maintenance of a defined healthy lifestyle.

Despite the effect of the COVID-19 pandemic on the awareness to get vaccinated, the rate of flu vaccination can be improved. HCWs know the benefits of the vaccine, but they do not get vaccinated. Education and awareness campaigns of health professionals must be strengthened to improve adherence to the flu vaccination programs. Indeed, the percentage of vaccinated health workers remained very low compared with the recommendation of the Ministry of Health that recommends a vaccination coverage of 75% as a minimum goal and above 95% as an optimal goal [10].

Concerning the limitations, first, the questionnaire had a low response rate. A possible selection bias was related to the interest to respond being limited to HCWs who have a positive opinion about flu vaccination. However, the vaccination registry gave us a picture of the real adherence to the vaccination programs during a long period (2013–2021). Second, there are some unknown information, such as the personal medical history of HCWs, that could have influenced the decision to get vaccinated or not. Further, the questionnaire was used to have an insight into the correlation of beliefs and opinions of HCWs and their willingness to receive the influenza vaccination in general and in the context of the COVID-19 pandemic.

The retrospective design, despite being a limitation to assess the effect of the campaign on the adherence to the vaccination program, allowed us to have a picture of the trend for 8 years. Furthermore, the information about HCWs’ vaccination status was based on vaccination records.

## 5. Conclusions

The onset of the COVID-19 pandemic strongly increased the attitude toward flu vaccination among HCWs. However, the rate of vaccinated HCWs remained low, and educational campaigns for health professionals must be strengthened to improve adherence to the flu vaccination programs. Additional training on vaccinations is necessary to improve HCWs knowledge and to address their concerns, which may lead to better vaccination uptake among this group.

## Figures and Tables

**Table 1:** Responses of HCWs concerning the knowledge and opinion about vaccinations.

	HCWs 2019–2020 *n* = 324	
Agree That	*n*	%
It is important to get vaccinated/to vaccinate to reduce/eliminate even serious diseases.	305	94.1
Serious side effects from the vaccine are hidden.	61	18.9
Serious adverse reactions to the vaccine are very rare.	233	72
By following healthy lifestyles, you can avoid diseases without the need to get vaccinated/to vaccinate.	38	11.6
If we stop getting vaccinated/to vaccinate, many diseases that are now rare could come back into circulation.	300	92.5
The disease is less dangerous than the vaccine itself.	54	16.6
Those who do not vaccinate/get vaccinated are blamed by National Health System operators.	74	22.8
National Health System operators are available to face doubts or perplexities about vaccinations.	235	72.6
National Health System operators are prepared and updated on vaccinations.	191	58.9
HCWs are familiar with Health Ministry recommendations about flu vaccination.	322	99.4
HCWs are aware that influenza is one of the diseases most easily contracted by health professionals.	288	89
HCWs are aware that influenza is one of the diseases most easily transmitted by health professionals.	305	94.1
HCWs are aware of the publication of the circular of the Ministry of Health, Prevention and control of influenza: recommendations for the 2020–2021 season, which expanded the range of the population for which vaccination is recommended and free.	225	69.5
HCWs are aware that among the recommendations for the prevention and control of influenza in the 2020–2021 season, “As regards the health and socio-health professions who operate in contact with patients […] vaccination is strongly recommended in the perspective of a legislative initiative that makes it mandatory.”	306	94.7

**Table 2:** Reasons to refuse flu vaccination.

	HCWs 2019–2020 *n* = 324	
	*n*	%
I am concerned about possible adverse events from the vaccine.	15	16.8
The vaccine is not effective in preventing the flu.	13	14.6
The vaccine is more dangerous than the virus itself.	13	14.6
I don’t know where the vaccination points are.	12	13.5
Vaccines are primarily an economic business of pharmaceutical companies.	10	11.2
I have little chance of contracting the disease.	9	10.1
If I contract the disease, the consequences will not worry me.	9	10.1
No specific reasons	8	9

**Table 3:** Influence of COVID-19 pandemic on HCW opinions on vaccination in 2019–2020 flu season.

	HCWs 2019–2020 *n* = 324	
	*n*	%
Information on the recent COVID-19 pandemic was obtained from Ministry of Health sources	291	69.5
HCWs consulted the ECDC and/or WHO web pages to obtain information on the recent COVID-19 pandemic.	134	41.4
The COVID-19 pandemic changed their opinion on the flu vaccination, deciding to get vaccinated.	99	30.5
HCWs intended to get vaccinated after the COVID-19 pandemic outbreak	110	33.9
HCWs did not want to get vaccinated/did not change their minds on flu vaccination after the SARS-CoV-2 pandemic outbreak.	5	1.6
HCWs had intention to get vaccinated if a vaccine against COVID-19 would be available.	114	35.2
HCWs would recommend anti-COVID-19 vaccination to the elderly and people with risk factors for severe disease.	126	40.8
HCWs would recommend both COVID-19 and flu vaccination to the elderly and people with risk factors for severe disease.	72	22.2
HCWs believe that influenza vaccination in healthcare workers for the 2020–2021 season could strengthen the workforce of the hospital in the event of a new wave of COVID-19, with fewer staff sick from the flu.	97	29.9
HCWS thought that the flu vaccination during the 2020–2021 season could facilitate the diagnosis and management of suspected cases of COVID-19, given the similar symptoms between COVID-19 and the flu.	93	28.7
Considering the SARS-CoV-2 pandemic, the vaccination coverage in healthcare workers will increase.	99	30.5

## Data Availability

The data presented in this study are available on request from the corresponding author. The data are not publicly available due to privacy hospital policy.

## Supplementary Materials

The following are available online at https://www.mdpi.com/article/10.3390/vaccines10060883/s1, Table S1: Sample characteristics of the whole sample of respondents to the questionnaire and in accordance with adherence to the vaccination in 2020; Table S2: Factors associated with the refusal of flu vaccination in 2020.
