# Supplementary material for: Attitudes of Healthcare Workers toward Influenza Vaccination in the COVID-19 Era
Source: Vaccines (Basel). 2022 May 31;10(6):883. doi: 10.3390/vaccines10060883 (PMC9231023; doi:10.3390/vaccines10060883)
Supplement: Supplementary file 1 [file vaccines-10-00883-s001.zip › vaccines-1688433-supplementary.pdf]

Table S1. Sample characteristics of the whole sample of respondents to the questionnaire and in accordance with adherence to the vaccination in 2020.

	**Total N=324**	**Did you get vaccinated during 2020 flu season? ***		
		**Yes n=103**	**No n=218**	**P**
**Age (mean, SD)**	48.1, 9.4	47.8, 9.9	48.1, 9.2	0.780
**Years of employment, n (%)**				0.188
≤5 years	31 (9.6)	8 (7.7)	23 (10.6)	
6-10 years	29 (9)	13 (12.6)	15 (6.9)	
>10 years	261 (80.9)	81 (78.6)	180 (82.6)	
Not declared	2 (0.6)	-	-	
**Gender§ , n (%)**				0.093
Male	65 (20.1)	26 (25.2)	38 (17.4)	
Female	258 (79.6)	76 74.5)	180 (82.6)	
Not declared	1 (0.3)	-	-	
**Professional role, n (%)**				0.001
Nurse	133 (41.3)	30 (29.1)	101 (46.3)	
Physician	99 (30.6)	48 (46.6)	51 (23.4)	
Others¥	25 (7.8)	9 (8.7)	15 (6.9)	
Health care professional	23 (7.1)	7 (6.8)	15 (6.9)	
Technician	22 (6.8)	5 (4.8)	17 (7.8)	
Nurse coordinator	20 (6.2)	4 (3.9)	16 (7.4)	
Not declared	2 (0.5)	-	-	
**Marital status, n (%)**				0.535
Married	218 (67.3)	69 (67)	147 (67.4)	
Single	72 (22.2)	23 (22.3)	48 (22.1)	
Divorced	30 (9.3)	11 (10.7)	19 (8.7)	
Widow	4 (1.2)	0	4 (1.8)	
**Setting, n (%)**				0.042
ED/ICU	62 (19.1)	18(17.5)	44 (20.2)	
Medical ward	102 (31.5)	39 (37.8)	61 (28)	
Surgical ward	37 (11.4)	7 (6.8)	30 (13.8	
Ambulatory care	83 (25.6)	30 (29.1)	53 (24.3)	
Non-clinical setting¥	23 (7.1)	3 (2.9)	20 (9.2)	
Not declared	17 (5.2)	-	-	

¥administrative, labs

§two workers did not respond

*Three subjects did not respond about flu vaccination in 2020

ED/ICU=Emergency Department/Intensive Care Unit

Table S2. Factors associated with the refusal of flu vaccination in 2020

	**p**	**Odds Ratio**	**95% C.I.**	
**Gender (reference: female)**	0.201	0.647	0.332	
**Years of work (reference <5 years)**	0.196			
6-10	0.079	0.310	0.084	10.145
>10	0.125	0.376	0.107	10.314
**Age**	0.379	10.017	0.979	10.056
**Role (reference: nurse coordinator)**	0.003			
Nurse	0.954	1.037	0.304	30.531
Physician	**0.033**	**0.254**	**0.072**	**0.893**
Health care professionals	0.253	0.419	0.094	10.861
Technicians	0.895	1.111	0.231	50.341
Others	0.580	0.660	0.152	20.867
**Setting (reference ED/ICU)**	0.017			
Medical ward	**0.007**	**0.333**	**0.149**	**0.745**
Surgical ward	0.625	0.756	0.246	2.322
Ambulatory	**0.016**	**0.336**	**0.138**	**0.818**
Non-clinical setting	0.864	1.139	0.255	5.083

ED/ICU=Emergency Department/Intensive Care Unit
